# A Case of Freeman-Sheldon Syndrome With Glaucoma Successfully Treated by Trabeculotomy Using the Kahook Dual Blade

**DOI:** 10.7759/cureus.76037

**Published:** 2024-12-19

**Authors:** Kihei Yoshida, Nariko Soga, Shuichiro Hirahara, Tsutomu Yasukawa, Miho Nozaki

**Affiliations:** 1 Department of Ophthalmology and Visual Science, Nagoya City University Graduate School of Medical Sciences, Nagoya, JPN

**Keywords:** distal arthrogryposis, freeman-sheldon syndrome (fss), kahook dual blade, minimally invasive glaucoma surgery (migs), primary open-angle glaucoma

## Abstract

This case study details a 41-year-old male patient with Freeman-Sheldon syndrome (FSS) who presented with ocular hypertension. The intraocular pressure (IOP) in his right eye progressively increased over time, leading to visual field loss, culminating in a diagnosis of juvenile-onset open-angle glaucoma (JOAG). Despite conventional medical therapies, adequate IOP control was not achieved, necessitating his referral to Nagoya City University Hospital. The patient underwent minimally invasive glaucoma surgery (MIGS), specifically trabeculotomy using the Kahook Dual Blade (KDB). Postoperative outcomes were favorable, with the IOP decreasing from approximately 40 to 10 mmHg and remaining consistently below 15 mmHg over a 27-month follow-up period. This case represents the first documented instance of glaucoma in a patient with FSS successfully managed with MIGS, highlighting the long-term efficacy and safety of this surgical intervention.

## Introduction

Freeman-Sheldon syndrome (FSS) is a rare congenital disorder characterized by arthrogryposis, also known as distal arthrogryposis (DA) type 2A, due to mutations in the MYH3 gene [1,2]. DA syndromes are a group of genetically induced contractures predominantly affecting the joints of the distal limbs, such as the hands, wrists, ankles, and feet [1-5]. FSS is considered the most severe form of DA. Reports of ocular complications associated with FSS are limited. Documented abnormalities include blepharophimosis, enophthalmos, hypertelorism, epicanthus, and strabismus [6-10]. While there is one report suggesting a potential association between FSS and juvenile glaucoma [11], no subsequent reports have confirmed this association, nor have other studies identified glaucoma as a complication of FSS. Herein, we describe the first adult case of juvenile-onset open-angle glaucoma (JOAG) in a patient with FSS and highlight the effectiveness of trabeculotomy using the Kahook Dual Blade (KDB), a minimally invasive glaucoma surgery (MIGS).

This article was presented at the 75th Annual Congress of Japan Clinical Ophthalmology held in Tokyo, Japan, between October 28 and October 31, 2021.

## Case presentation

**Figure 1:**
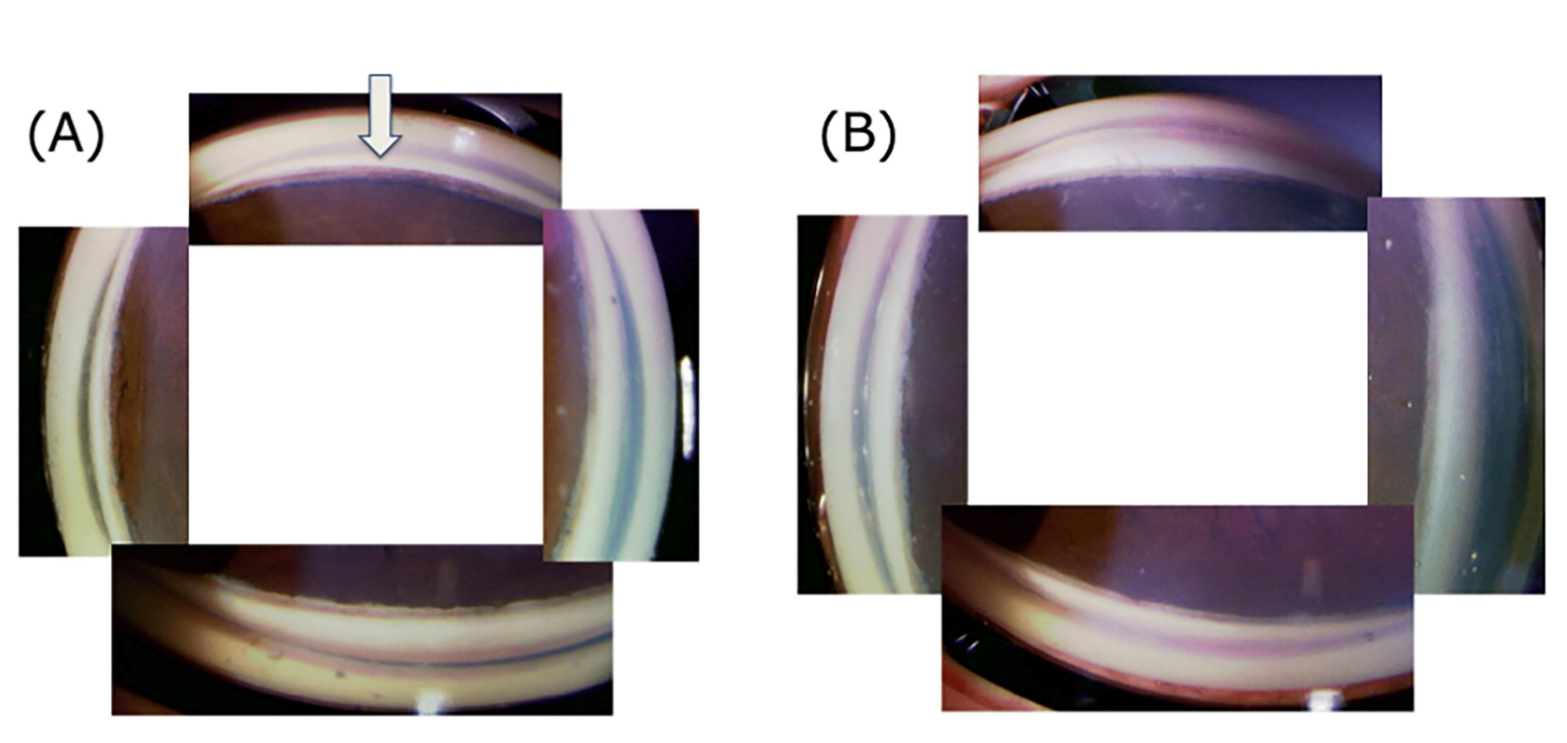
Gonioscopic images of the anterior chamber angle. (A) Gonioscopic view of the right eye, showing pigment deposition on the trabecular meshwork (indicated by the arrow). The angle is wide open, with visible structures, including the Schwalbe’s line, trabecular meshwork, and scleral spur. (B) Gonioscopic view of the left eye, showing a clear trabecular meshwork without significant pigment deposition. The angle is similarly wide open, with no abnormal findings.

A 41-year-old male with a known diagnosis of FSS was referred to our ophthalmology clinic for the management of ocular hypertension and dry eye symptoms. His systemic history included talipes equinovarus, scoliosis, and arthrogryposis. The diagnosis of FSS in this case was made based solely on clinical findings, as genetic testing was not performed. Notably, he exhibited no signs of intellectual disability. Initially, the patient was managed at a local clinic, where he was treated with latanoprost in both eyes. However, despite this treatment, the intraocular pressure (IOP) in his right eye rose to 38 mmHg, accompanied by progressive visual field deterioration. Subsequent intensive medical therapy, including carteolol hydrochloride, brimonidine, ripasudil hydrochloride hydrate, pilocarpine hydrochloride, and oral acetazolamide, as well as selective laser trabeculoplasty (SLT), failed to achieve adequate IOP control. The patient was then referred to Nagoya City University Hospital for advanced glaucoma management. He was asymptomatic and had no history of steroid use. On examination, his corrected visual acuity was 20/40 in the right eye and 20/16 in the left eye. IOP measurements were 36 mmHg in the right eye and 11 mmHg in the left eye. Central corneal thickness (CCT), measured using CASIA 2® (TOMEY Corp., Nagoya, Japan), was 518 μm in the right eye and 502 μm in the left eye. Axial length (AL), measured with IOLMaster 500® (Carl Zeiss Meditec, Dublin, CA), was 25.1 mm in the right eye and 23.6 mm in the left eye. Corneal endothelial cell density was 2,245 cells/mm² in the right eye, indicating no significant endothelial dysfunction. Clinical evaluation revealed blepharoptosis and horizontal nystagmus. Anterior segment evaluation was unremarkable, and the angle was wide open in both eyes. Pigment deposition was noted on the trabecular meshwork of the right eye (Figure 1).

**Figure 2:**
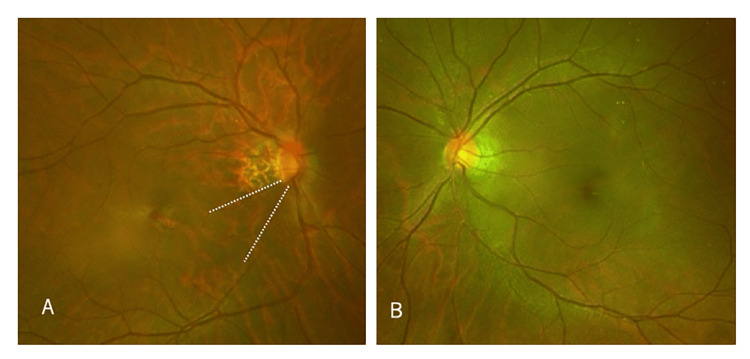
Funduscopic images. (A) The right eye shows a nerve fiber layer defect in the lower temporal quadrant, highlighted by dotted lines. (B) The left eye shows no evidence of nerve fiber layer defects (NFLDs).

Funduscopic examination revealed a cup-to-disc ratio of 0.8 in the right eye and 0.6 in the left eye, with a nerve fiber layer defect in the inferior temporal quadrant of the right eye (Figure 2).

**Figure 3:**
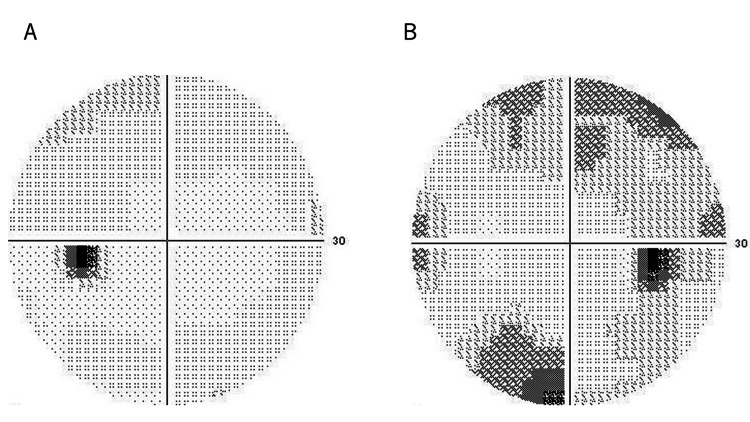
Visual field-testing results from the Humphrey Field Analyzer (HFA). The left eye shows a normal visual field without defects (A). The visual field of the right eye demonstrates an upper Bjerrum scotoma (B), indicative of glaucomatous visual field loss.

Visual field testing using the Humphrey® Field Analyzer (HFA) revealed an upper Bjerrum scotoma in the right eye, consistent with glaucomatous damage. The mean deviation (MD) in the right eye was measured at -5.62 dB, indicative of early-stage glaucoma according to the Hodapp-Parrish-Anderson classification system (Figure 3).

**Figure 4:**
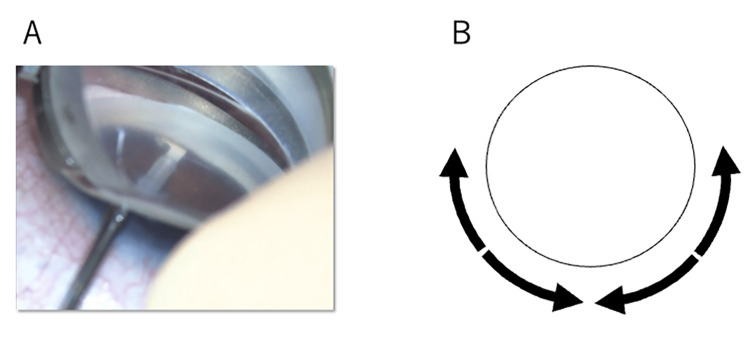
Intraoperative images of excisional goniotomy performed with the Kahook Dual Blade. (A) The excisional goniotomy was performed using the Kahook Dual Blade, targeting the inferior trabecular meshwork through two side-port incisions at the 2 o’clock and 10 o’clock positions. (B) The excisional goniotomy covered all areas of the trabecular meshwork except at the 6 o’clock position, with a focus on the temporal-inferior-nasal region.

Optical coherence tomography (OCT) imaging could not be obtained due to the patient’s horizontal nystagmus. Preoperatively, the right eye exhibited an IOP of 40 mmHg, despite maximal medical therapy. Surgical intervention with MIGS was undertaken. Specifically, an excisional goniotomy was performed using the Kahook Dual Blade (New World Medical, Rancho Cucamonga, CA), targeting the inferior trabecular meshwork through two side-port incisions at the 2 o’clock and 10 o’clock positions (Figure 4).

**Figure 5:**
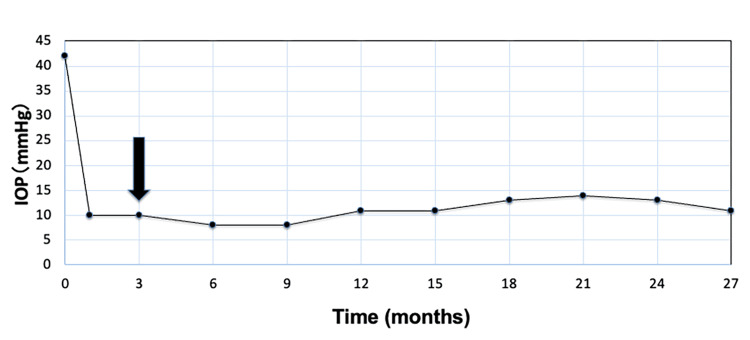
Intraocular pressure (IOP) follow-up during the 27 months after surgery in the right eye. The graph illustrates the IOP trend in the right eye following excisional goniotomy with the Kahook Dual Blade. Postoperatively, the IOP decreased to 10 mmHg. At three months after surgery, dorzolamide-timolol fixed combination therapy was initiated, as indicated by the arrow on the graph. The IOP remained stable below 15 mmHg throughout the 27-month follow-up period.

The procedure was uneventful and resulted in a reduction of IOP to 10 mmHg postoperatively. No complications were reported. Over a follow-up period of 27 months, IOP in the right eye remained stable below 15 mmHg with the adjunctive use of a dorzolamide-timolol fixed combination (Figure 5).

## Discussion

The case presented here is the first documented instance of JOAG in an adult patient with FSS, successfully managed with trabeculotomy using KDB. The underlying pathophysiology connecting FSS with glaucoma remains speculative, as FSS is primarily associated with skeletal and craniofacial abnormalities due to mutations in the MYH3 gene. While some ocular abnormalities in FSS have been documented, such as blepharophimosis, enophthalmos, hypertelorism, epicanthus, and strabismus [6-10], reports specifically linking FSS to glaucoma are extremely limited.

Previous literature on FSS and glaucoma is scarce. Mueller and Meyer [11] reported a potential association between distal arthrogryposis type 2A (FSS) and juvenile glaucoma. However, this report lacked follow-up details, including IOP measurements, progression of the cup-to-disc ratio, and visual field loss. Therefore, it remains unclear whether the patient described in that study developed glaucoma or simply exhibited an enlarged cup-to-disc ratio without progressive glaucomatous changes. Our case is the first to provide a confirmed diagnosis of glaucoma in an adult FSS patient, contributing a new perspective on the spectrum of complications in FSS.

Further insights can be drawn from related conditions. Sahni et al. [12] described ocular findings in distal arthrogryposis type IIB, a condition related to FSS. The reported ophthalmic manifestations included hypertelorism, bilateral ptosis, ophthalmoplegia, astigmatism, and strabismus, which are also seen in FSS. Additionally, they noted thickening of the central cornea, reduced axial length, and choroidal folds. The thickening of the central cornea may contribute to an overestimation of IOP; however, it may also reflect biomechanical alterations that influence aqueous outflow resistance. Reduced axial length, in combination with choroidal folds, may suggest anatomical crowding in the anterior chamber angle, potentially impairing aqueous outflow.

However, in our case, the patient exhibited normal corneal thickness and a long axial length, findings that do not align with those previously reported. This discrepancy underscores the potential heterogeneity in the ocular manifestations of FSS.

Abnormal collagen deposition has been suggested as a possible mechanism contributing to impaired aqueous outflow in FSS [11]. In our case, we believe that collagen abnormalities may have played a role in the development of glaucoma, despite the lack of overt gonioscopic anomalies beyond trabecular meshwork pigmentation. This finding raises the possibility that structural anomalies in the trabecular meshwork or other outflow pathways might be subtle or localized, and not easily detectable by conventional examination methods. Further investigations, including pathological assessments of angle tissues, could provide valuable insights into the underlying mechanisms of glaucoma in FSS patients. Unfortunately, due to the retrospective nature of this study, a comprehensive pathological assessment of the trabecular meshwork was not performed. In the event of recurrence or further complications, obtaining a biopsy of angle tissues could provide valuable insights into the underlying pathology and enhance our understanding of the disease mechanism in FSS. In this case, although SLT was ineffective, trabeculotomy was chosen as the preferred treatment due to the early stage of glaucomatous visual field impairment and the diagnosis of JOAG, which is considered an ideal indication for this procedure. Several studies have reported the long-term outcomes of trabeculotomy for developmental glaucoma. Akimoto et al. [13] concluded that trabeculotomy ab externo remains effective over time. Similarly, Ikeda et al. [14] demonstrated its efficacy, noting that the success probability for JOAG was lower than that for infantile glaucoma. Although outcomes for JOAG were slightly less favorable, the overall results support the efficacy of trabeculotomy for developmental glaucoma. These findings reinforce that the success of MIGS using the Kahook Dual Blade in this case is consistent with the reported evidence, particularly for juvenile-onset glaucoma. The success of MIGS using the KDB also supports the hypothesis that resistance at aqueous outflow due to abnormal collagen deposition might be the pathogenesis of IOP elevation in the eyes of FSS.

## Conclusions

The successful reduction of IOP and stabilization of glaucoma in this patient with FSS through MIGS using the Kahook Dual Blade suggests that this approach could be effective for similar cases. Given the potential for ocular complications in FSS, regular ophthalmic monitoring, including IOP measurements and detailed optic disc assessments, is recommended. Further studies and case reports are needed to establish a clearer understanding of the prevalence and management of glaucoma in FSS.
